# Tuberculosis incidence in patients treated with secukinumab – a real-world study in Brazil

**DOI:** 10.1186/s42358-025-00496-x

**Published:** 2025-12-29

**Authors:** Felipe Merchan Ferraz Grizzo, Sineida Maria Berbert Ferreira, Maria Victória Suárez Restrepo, Anber Ancel Tanaka, André Vicente Esteves de Carvalho, Roberto Ranza, Felipe Thies, Juliana Cabrera Garrido

**Affiliations:** 1 Rheumatology Service, Parana Medical Research Center, PMRC, Av. Carlos Correa Borges, 933 - Zona 05, Maringá, Paraná 87060-000 Brazil; 2 Dermatology Service, Paranaense Center for Dermatology Studies, CEPED, Maringá, Paraná Brazil; 3 Dermatology Service, Hospital Oswaldo Cruz, São Paulo, São Paulo Brazil; 4 Dermatology Service, Mackenzie Evangelical University Hospital, São Paulo, São Paulo Brazil; 5 Dermatology Service, Hospital Moinhos de Vento, Porto Alegre, Rio Grande do Sul Brazil; 6 Rheumatology Service, Federal University of Uberlândia, Uberlândia, Minas Gerais Brazil; 7 Novartis Biosciences S.A, São Paulo, São Paulo Brazil

**Keywords:** Secukinumab, Psoriasis, Axial spondyloarthritis, Psoriatic arthritis, Tuberculosis, Real-world

## Abstract

**Background:**

Tuberculosis (TB) remains a global health challenge, especially in low- and middle-income countries. Tumor necrosis factor alpha (TNFα) plays a crucial role in the immune response to TB. TNF inhibitors were the first biologic agents approved for treating chronic inflammatory diseases such as psoriasis (PsO), axial spondyloarthritis (axSpA) and psoriatic arthritis (PsA). Despite their efficacy, TB risk is a concern. The development of new biological therapies, as IL-17 inhibitors, has improved these diseases’ management; however, data regarding TB risk remain scarce. This study assessed TB incidence in Brazilian patients with PsO, axSpA and PsA treated with secukinumab.

**Methods:**

This real-world retrospective study included Brazilian patients treated for at least 24 months, aged ≥ 18 years, without confirmed tuberculosis diseaseTBD. Data were extracted from medical charts. Descriptive analyses used mean or median, standard deviations, and quartiles for continuous variables and absolute frequencies and percentages for categorical variables with 95% confidence intervals calculation, as applicable.

**Results:**

152 participants were included, with a mean follow-up of 40.1 months, predominantly PsA (n = 90) and white (84.6%), with an average age of 52 years (IQR: 42–61) and a slightly male predominance (50.7%). Hypertension for PsA (11.9%) and PsO (16.3%), and fibromyalgia for axSpA (16.0%) patients were the most common comorbidities. PsO patients had the longest disease duration (184.6 months). Secukinumab loading doses were administered to 141 patients, with 78% receiving 300 mg. Maintenance doses were 300 mg for 77% of the patients. Secukinumab treatment was discontinued in 18 patients (11.8%). On average, patients were on 3.35 concomitant medications before secukinumab, dropping to 2.26 afterward, with methotrexate being the most used medication before secukinumab. No TBD cases were recorded. Tuberculosis infection (TBI) testing showed some positive results, and preventiveantibiotic therapy was administered as needed. Most patients remained negative for TBI after treatment. No safety-related information was detailed.

**Conclusion:**

This real-world evidence study demonstrated that no TBD cases were recorded even after receiving secukinumab, and most patients were negative for TBI. Further studies are recommended to enhance the knowledge of TB prevention among patients with axSpA, PsO, and PsA.

## Introduction

Tuberculosis (TB) is an infectious disease, transmitted via aerosol, caused by *Mycobacterium tuberculosis*, that remains a significant global health challenge, affecting approximately one-quarter of the world’s population and causing over 1.5 million deaths annually [1]. TB is particularly prevalent in low to middle-income countries, with Brazil identified as one of the high-burden countries [2, 3]. According to the World Health Organization (WHO), Brazil is one of the 30 countries with high TB burden, with the disease being the leading cause of death by a single infectious agent worldwide [1]. In 2023, the incidence was 37,0 per 100,000 inhabitants, with a higher prevalence of cases in the Northern region of Brazil [4].

In immunocompetent individuals, TB is initially managed by their immune defenses, leading to an asymptomatic state referred to as tuberculosis infection (TBI) [5, 6]. Tumor necrosis factor alpha (TNFα) plays crucial role in the immune response to TB infection and prevention of TBI reactivation by being responsible for macrophages activation, forming and maintaining granulomas, apoptosis initiation and promoting CXCR3 + T cells migration [7]. On the other hand, the use of TNF inhibitors (TNFi), that are effective for immune-mediated or inflammation-related diseases can disrupt this protective mechanism, leading to an increased risk of TB infection or reactivation of TBI as demonstrated in in vitro [8] and in clinical research studies that reported a four-fold increase in the risk of TB infection [9–12]. Besides, a systematic review and meta-analysis suggested that the risk of TB was doubled in patients with any condition treated with TNFi. This finding emphasizes that when considering. TNFi as a treatment option, the increased risk of TB should be part of the treatment decision-making process [12].

Psoriasis (PsO), axial spondyloarthritis (axSpA) and psoriatic arthritis (PsA) are chronic inflammatory, immune-mediated diseases. The global prevalence of AxSpA ranges from 0.3 to 1.4% [13], while for PsO it is approximately 2 to 3% [14]. PsA occurrs in approximately 0.1 to 1% of the population, with prevalence rates varying by geographical region [15]. In Latin America, the prevalence of PsO and axSpA closely aligns with global rates, affecting around 2.5% (PsO) [16] and between 0.02 and 0.8% (axSpA) [17] of the population. PsA is less common in Brazil, with an estimated prevalence of 24.4 cases per 100,000 patients receiving follow-up care in the public health system (SUS) [18].

In the fields of rheumatology and dermatology, TNFi are the most commonly used immunosuppressive agents for treating these conditions [19, 20]. Over the past decades, advancements in understanding the pathogenesis of chronic inflammatory diseases, including axSpA, PsA, and PsO, have led to significant evolution in treatment strategies [21, 22]. Secukinumab is a human monoclonal antibody that targets interleukin-17 A (IL-17 A), a key inflammatory cytokine in several chronic immune mediated conditions, including axSpA, PsA and PsO [23]. Despite clinical trials and real-world studies have shown promising results for secukinumab, suggesting no significant impact on the progression or reactivation of primary TB infection, the available evidence remains limited [24–26].

Currently, there is no local data on the incidence of TB among biologic-treated patients with axSpA, PsO, and PsA. Hence, the purpose of this study was to assess the incidence of TB in Brazilian first biologic naïve (those with no prior exposure to biologic drugs) and secondary-naïve (those with previous biologic exposure with a wash-out period of at least 3 months before the index date) patients with axSpA, PsO and PsA, treated with secukinumab.

## Methods

### Study design

This was a real-world retrospective, non-interventional study in Brazil to evaluate the exposure-adjusted incidence of TBD and TBI among biologic-naïve (those with no prior exposure to biologic drugs) and secondary-naïve (those with previous biologic exposure with a wash-out period of at least 3 months before the index date) patients with axial spondyloarthritis (axSpA) and psoriatic arthritis (PsA) and psoriasis (PsO) who underwent secukinumab treatment for at least 24 months [27].

Retrospective data were collected in public and private health care settings from 14 sites distributed in different geographic regions across Brazil. Data extracted from patient chart reviews at the participating hospital centers were entered into an electronic case report form designed to capture all relevant information (demographic, diagnosis, secukinumab treatment, comorbidities, treatment history and tuberculosis history). The study was conducted in compliance with the guidelines for good pharmacoepidemiology practices (GPP) of the International Society for Pharmacoepidemiology. All participating sites had ethical approval for the study from their respective local Ethics Committees and the study followed the principles in the Declaration of Helsinki and national ethical requirements established in the Resolution n 466/2012.

### Study population and study setting

Participants were primarily identified with axSpA, PsA, and PsO diagnosis according to the International Classification of Diseases (ICD-10) code M45, M07, and L40, respectively. Alternatively, the written diagnosis from applicable departments were also used for patient screening. Patients classified under the axSpA + PsA and axSpA + PsO subgroups presented clinical and/or imaging features consistent with axial involvement and were categorized separately from the broader PsA group based on medical judgment aligned with ASAS classification criteria to enhance analytical granularity.

**Fig. 1:**
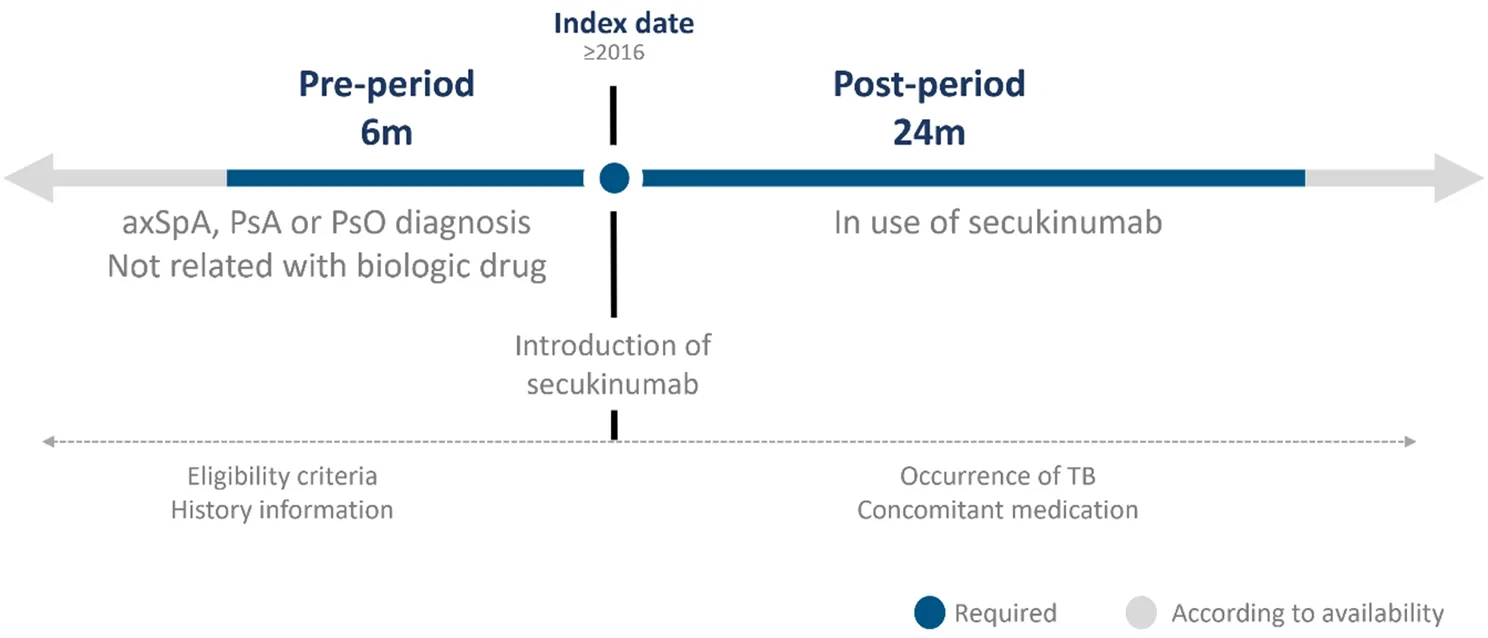
Summary of participant study period. axSpA, axial spondyloarthritis; PsA, psoriatic arthritis; PsO, psoriasis; TB, tuberculosis

Patients aged 18 years or older at secukinumab initiation, who were diagnosed with axSpA, PsA, or PsO, regardless of severity, and treated with secukinumab for at least 24 months, from January 2016 to September 2021, were included in the study. Only biologic-naïve patients or secondary-naïve patients were included to decrease carryover (residual) effect of other biologic therapy on the occurrence of TB [28–30]. In addition, patients needed a minimum of six months medical history covering before and 24 months after secukinumab initiation (Fig. 1).

Patients were excluded from the study if they met any of the following criteria: lack of access to medical charts, use of a non-approved secukinumab dosing regimen, a confirmed history of TBD or Mendelian susceptibility to mycobacterial diseases before secukinumab use, and treatment with systemic corticosteroids at immunosuppressive doses (prednisone > 10 mg or equivalent) for at least two consecutive weeks, or other concomitant immunosuppressive drugs during the study period.

The study was conducted across a diverse range of geographic regions in Brazil, enhancing the generalizability of the findings. Participating centers were located in the North and Northeast (Salvador–BA, Recife–PE), Central-West (Anápolis–GO), Southeast (São Paulo–SP, São José do Rio Preto–SP, Ribeirão Preto–SP, Campinas–SP, Uberlândia–MG), and South (Porto Alegre–RS, Curitiba–PR, Maringá–PR, Florianópolis–SC). This broad national coverage ensures representation from multiple healthcare settings and regional populations, contributing to the robustness and external validity of the study outcomes.

### Statistical analysis

Sociodemographic data and relevant clinical information were recorded, including age, gender, ethnicity and comorbidities that were stratified by disease subgroup (axSpA, PsA, PsO, axSpA + PsA, axSpA + PsO) and concomitant medication. Clinical characteristics (i.e.: comorbidities) were determined at the time of introduction of secukinumab (index). The disease duration (months) for all patients was obtained from the time of the last diagnosis until the enrollment date.

Descriptive analysis was performed on the collected data. Continuous variables were summarized using means and standard deviations (SD) or medians and inter quartile range (IQR) (25th to 75th percentile), along with minimum and maximum values. Categorical variables were summarized using absolute frequencies and percentages, calculated over the number of patients with available data. 95% confidence intervals (CIs) were calculated as applicable. Changes over time were assessed by comparing two periods (e.g., mean change in score or change in disease severity category). Data computations were made using R^®^ version 4.4.0 (R Core Team). Any missing data were reported, and no data imputation was performed.

## Results

**Fig. 2:**
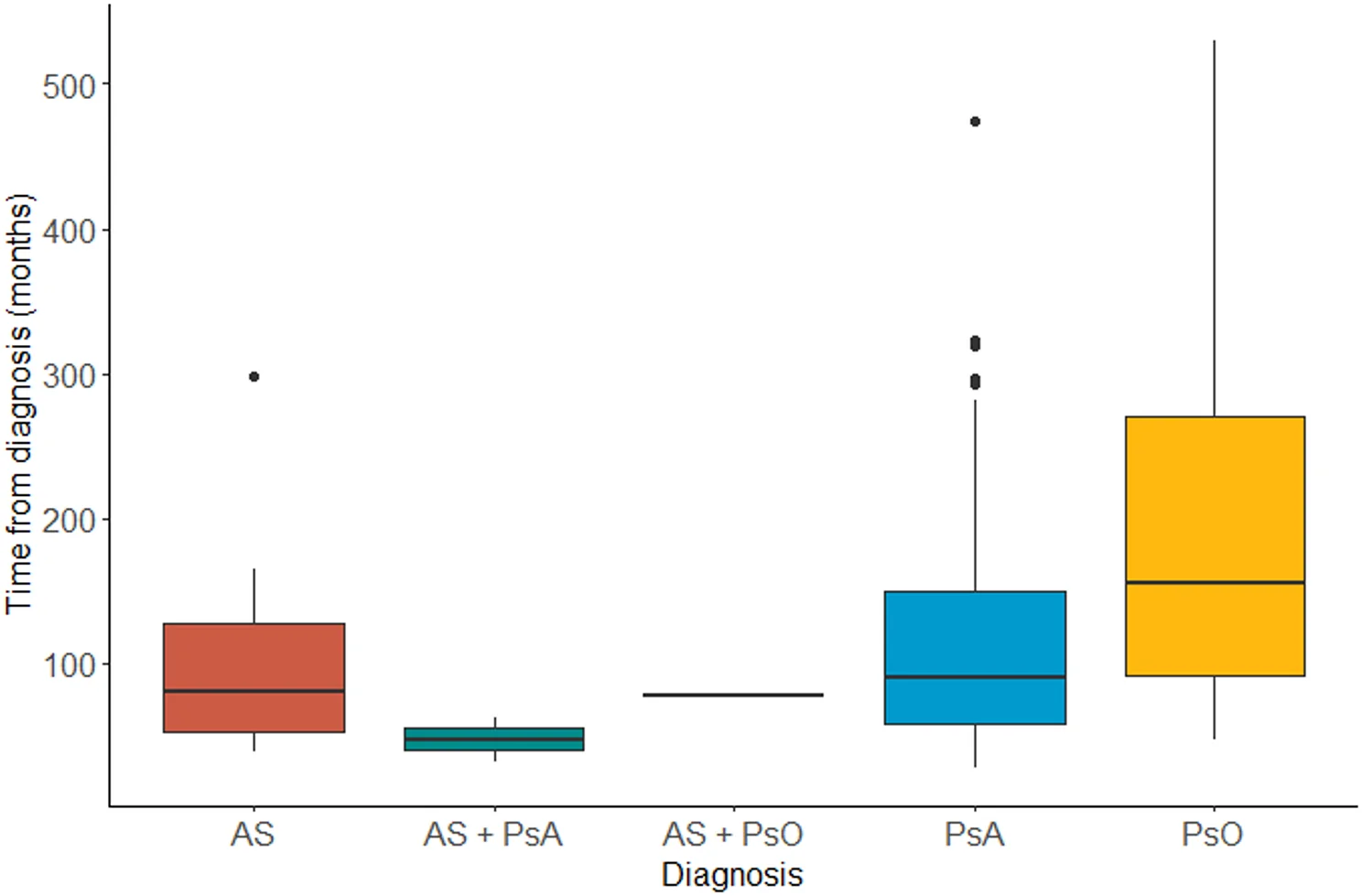
Boxplots of time (in months) from most recent diagnosis to enrollment by diagnosis

A total of 225 participants were recruited, of whom 152 participants were included in the study. The remaining 73 participants were excluded due to meeting at least one exclusion criteria. The study population was categorized into five disease subgroups: axSpA, PsA, PsO, axSpA + PsA, axSpA + PsO. Most participants belonged to the PsA subgroup (*n* = 90; 59.2%) with a mean (SD) duration of 113.7 (80.7) months. In contrast, the axSpA (*n* = 30; 19.7%) and PsO (*n* = 29; 19.1%) subgroups had mean (SD) durations of 93.6 (55.3) and 184.6 (113.7) months, respectively (Table 1). A boxplot depicting disease duration by the disease sub-group is presented in Fig. 2.

The overall mean (SD) age of the patients was 51.8 (13.2) years, and the proportion of male (50.7%) and female (49.3%) patients was similar among the disease subgroups (Table 1). Most of the patients were of white ethnicity (84.6%), followed by a mixed ethnicity (11.4%).

### Comorbidities by disease subgroups and concomitant medications

Among the 152 patients, 99 (65.1%) had pre-existing comorbidities before starting secukinumab treatment; of these, 24 (80%) were in the axSpA subgroup, 57 (63.3%) in the PsA subgroup, and 16 (55.2%) in the PsO subgroup. Prior to the initiation of secukinumab, a total of 239 comorbidities were documented: 50 in the axSpA subgroup, 135 in the PsA subgroup, and 49 in the PsO subgroup. Hypertension was the most frequently reported comorbidity across the entire study population and within each subgroup, except for axSpA, where the most frequently reported comorbidity was fibromyalgia. The distribution of comorbidities by diagnosis is illustrated in Table 2.

Table 3 outlines the use of concomitant medications before and after the start of secukinumab treatment. Before secukinumab treatment, participants received a total of 482 concomitant medications, which decreased to 120 medications after starting the treatment. On average, patients were on 3.35 concomitant medications each before secukinumab, dropping to 2.26 afterward.

The main reason for prescribing these medications was to target axSpA, PsA, and PsO, accounting for 123 participants (85.4%) before secukinumab and 17 participants in use of these medications (32.0%) after secukinumab (Table 3). Before starting secukinumab, the most used medication for axSpA, PsA, and PsO were oral methotrexate (74 participants, 60.2%). Overall, Disease-Modifying Antirheumatic drug (DMARDs) was the most used class of medication prior secukinumab (Table 3). Corticosteroid use reported in the study encompassed both systemic and topical formulations.

### Secukinumab use and tuberculosis

All participants underwent secukinumab treatment for at least 24 months, with a mean (SD) treatment duration of 39.3 (13.8) months. Among 152 patients, a loading dosage was prescribed for 141 (92.8%) participants for 5 weeks; of them, most (*n* = 110; 78.0%) received 300 mg secukinumab and 31 (22.0%) received 150 mg secukinumab once every week. For the maintenance dosage, 117 patients (77.0%) received 300 mg secukinumab and 35 patients (23.0%) received a dosage of 150 mg secukinumab every 4 weeks. The dosage of secukinumab was switched among 14 patients (9.2%) and increased from 150 mg to 300 mg once every 4 weeks (data not shown). Secukinumab treatment was interrupted in a total of 18 patients (11.8%). The mean (SD) duration of interruption was 3.8 (3.1) months, ranging from 1.1 to 13.0 months. No treatment interruptions were reported due to safety concerns directly associated with secukinumab. Immunobiological medications were used in 72 patients (47.4%) before the initiation of secukinumab (Table 4).

Tuberculosis screening included both systemic and imaging-based assessments, with patients undergoing a combination of purified protein derivative (PPD) tests, chest radiographs, and, in fewer cases, Interferon-Gamma Release Assays (IGRA), such as the QuantiFERON-TB Gold test. Among those tested prior to secukinumab initiation, 97.9% received PPD testing, 64.3% underwent chest radiography, and 2.1% received IGRA. In the post-initiation group, 58.3% underwent PPD testing and 8.3% received IGRA. No TBD cases were recorded in any of the participants, as per disease subgroups, when using secukinumab for at least 24 months (Table 5). Among the participants, 3 (10.7%) with axSpA, 19 (21.3%) with PsA, and 9 (32.1%) with PsO tested positive for TBI prior to the initiation of secukinumab. Of the 32 participants who tested positive for TBI, 21 received preventive antibiotic therapy. Among them, 2 (66.7%) had axSpA, 14 (73.7%) had PsA, 4 (44.4%) had PsO and 1 (100%) had axSpA + PsA. Following the initiation of secukinumab, 3 participants with PsA and 2 with PsO tested positive for TBI, and all received preventive antibiotic therapy. One participant (8.3%) with PsA, who was not tested for TB prior to secukinumab initiation, also received preventive antibiotic therapy (based on clinician’s decision) (Table 5).

Among the 116 patients who tested negative for TBI prior to the initiation of secukinumab treatment, 113 (97.4%) remained negative for TBI, while 3 (2.6%) converted from negative to positive for TBI following secukinumab treatment. There are no available data on the treatment of these patients for TBI. Of the 32 patients who tested positive for TBI before starting secukinumab, 30 (93.8%) converted from positive to negative for TBI after secukinumab treatment, whereas 2 (6.2%) remained positive for TBI.

## Discussion

This retrospective, non-interventional real-world evidence study in Brazil aimed to evaluate the incidence of TB among biologic-naïve and secondary biologic-naïve patients with axSpA, PsO, and PsA treated with secukinumab, addressing the lack of clarity regarding whether the choice of treatment affects the incidence of TB in these patients. According to a systematic review and meta-analysis of randomized clinical trials and observational studies published up to July 2015 on the harms of 13 target immunomodulators in autoimmune diseases, etanercept might be associated with lower TB rates than adalimumab and infliximab; however, the evidence supporting this is weak, and further research is necessary to establish a causal link [31]. Nonetheless, a study from a Brazilian registry of biological therapies used for rheumatic diseases from 2009 to 2013 reported a relative risk for TBD of 4.38 in patients using adalimumab for rheumatoid arthritis [32]. The study also calculated the incidence of TBD per 1000 patient-years in rheumatoid arthritis patients: 2.86 for those using any TNF inhibitor, 4.43 for adalimumab users, 1.82 for infliximab users, 1.92 for etanercept users, and no cases for patients using other biologics (including rituximab, abatacept, and tocilizumab) [32].

A comprehensive pooled analysis, which included 28 clinical trials of secukinumab involving a total of 12,319 patients (8,819 with PsO, 2,523 with PsA, and 977 with axSpA), reported no evidence of TB. Besides, the analysis concluded that reports of TBI following treatment were uncommon, supporting the use of secukinumab in chronic systemic inflammatory conditions [33]. However real-world incidence of TBD in Brazil among secukinumab-treated patients remains unknown [25, 34]. Given the high burden of TB in Brazil, it is advised that TB infection be considered and investigated in patients before initiating biological therapy [35]. Understanding the incidence of TBD in axSpA, PsO, and PsA patients during treatment could aid in the planning and implementation of national TB control policies [36].

Since secukinumab was first approved by the Brazilian National Regulatory Agency (ANVISA) for these diseases by the end of 2015, the study only included patients who were exposed to secukinumab from January 2016 onwards. Patients initiated secukinumab treatment after its introduction in January 2016 and remained on the drug for at least 24 months. A higher number of participants presented with PsA (*n* = 90), followed by axSpA (*n* = 30) and PsO [30]. A small number of patients presented with axSpA + PsA (*n* = 2) and axSpA + PsO (*n* = 1). Patients with PsO had the longest disease duration (184.6 months) since the diagnosis, compared with patients with axSpA (93.6 months) and patients with PsA 113.7 months since the diagnosis. Overall, the mean age of the patients was 51.8 years. Typically, the number of PsO diagnoses is three times higher compared to PsA diagnoses [37]. However, in this study, a higher percentage of patients were diagnosed with PsA than with PsO, probably because secukinumab received its ANVISA approval for rheumatology indications before it was approved for dermatology uses [38].

A total of 99 participants (65.1%) had 239 comorbidities and the most common comorbidity for PsA and PsO subgroups was hypertension. The findings are in line with results from other studies that also reported hypertension as the most common comorbidity for a population with similar diagnoses [39–42]. In contrast, for the axSpA subgroup, fibromyalgia was the most frequently reported comorbidity (16%). Corroborating this finding, a recent meta-analysis revealed that although there is a significant variation in fibromyalgia prevalence estimates in axSpA patients, fibromyalgia is a common comorbidity in those patients, experienced by approximately one in six axSpA patients [43]. Furthermore, rheumatic diseases are associated with several comorbidities due to their systemic inflammatory component [44]. PsA, PsO and axSpA have all been associated with an increased risk for cardiovascular outcomes [45–47], and obesity has been consistently identified as a risk factor for psoriatic disease [48].

Regarding the history of prior systemic therapy exposure, clinical trials reported that only 12–29% of the participants had previously received biological therapy [49]. However, in this study, 65.0% of the participants received DMARDs, with 60.2% being under methotrexate, and 58.5% received immunobiological therapy before starting secukinumab. Oral methotrexate was the most utilized medication, as expected since it is the first-line treatment for PsA according to Brazilian guidelines [50]. These differences highlight the importance of RWE studies to complement clinical trials results as RWE approaches involve patients with diverse backgrounds.

After starting secukinumab treatment, there was a reduction in the number of concomitant medications in use, with the most used class of medication being corticosteroids. Similarly, a recent prospective real-world study evaluating the proportion of patients with PsA, axSpA, or PsO who benefit from the continuation of treatment with etanercept, a TNFi, reported that while there was a reduction in concomitant glucocorticoid use across all patient populations, half of patients with axSpA and PsA remained on long-term, low-dose glucocorticoid throughout the study [51]. As patients with these chronic diseases can experience common symptoms due to inflammation and pain, a reduction on concomitant medication during the use of secukinumab suggests that the therapy is providing an adequate disease management. Concomitant glucocorticoid use is reported for many studies that report disease conversion from positive to negative but also adverse effects. Brazilian guidelines suggest that glucocorticoids should be used with caution and highlight the need of an appropriate disease management especially due to the adverse effects caused by the use of these medications [50, 52, 53].

The mean (SD) duration of secukinumab treatment was 39.3 (13.8) months and loading dose was prescribed for 141 patients (92.8%). Of them, 31 (22.0%) received a dose of 150 mg, while 110 (78.0%) received a dose of 300 mg once in every one week. Similarly, for the maintenance doses, 117 patients (77.0%) received a dose of 300 mg and 35 patients (23.0%) received a dose of 150 mg once in every 4 weeks. Secukinumab interruption was reported for 11.8% of the participants, which is lower than the rates reported in other real-world studies that was around 20%, mostly due to lack of effectiveness [54, 55]. On the other hand, a four-year real-life study including 685 patients with PsA stated that secukinumab proved to be safe and effective with patients achieving a notable drug retention [56]. Besides, data from randomized trials and post-marketing surveillance studies have shown that secukinumab has a favorable safety profile with fewer adverse events and a low treatment discontinuation rate [57].

There is evidence suggesting that the depletion of IL-17–producing T cells is not directly associated with the progression of *M. tuberculosis* infection [58, 59]. Supporting these mechanistic data, clinical studies targeting IL-17 pathways with biologics have reported few cases of TBD or TBI reactivation. One of these studies compiled data from five randomized, double-blind, placebo-controlled, phase 3 secukinumab studies in 2044 subjects with moderate to severe plaque psoriasis and concluded the absence of TB reactivation in secukinumab clinical studies [26]. Similarly, a systematic review of the literature compiling 3 controlled trials of PsA and 2 trials of AS respectively, enrolling a total number 1045 and 620 patients treated with secukinumab reported no cases of TB reactivation [25]. Another study pooled data from 28 clinical trials and a postmarketing safety surveillance in 8,819 patients with PsO, 2,678 with PsA and 1,140 with axSpA found incidence of opportunistic infections such as TB, bronchopulmonary aspergillosis, cytomegalovirus gastroenteritis, and gastrointestinal candidiasis, of 0.19/100 patient-years for PsO, 0.18/100 patient-years for PsA, and 0.14/100 patient-years for axSpA; TBD or TBI were rare [57]. IL-17 is involved in immune response, recruiting cell to infection sites, but does not play a central role in granuloma formation which corroborates with the above results suggesting that IL-17 inhibitors are not related to TB infection even for patients with TB history [60].

In this study there was no incidence of TBD among participants and out of the 116 participants who were tested negative for TBI prior to secukinumab treatment, 3 of them (2.6%) had a shift from negative to positive for the presence of TBI after secukinumab treatment, and all of them received preventive antibiotic therapy. Although specific time intervals between baseline and follow-up tests were not consistently documented, patients without prior TB testing had their negative status confirmed clinically by the treating physician, ensuring appropriate safety monitoring throughout the study. Despite Brazil is considered an endemic country for TB, the study demonstrated a low conversion rate to TBI and the absence of TB infection, highlighting the safety of secukinumab even in areas with a high TB burden. These findings are in line with existing literature, which suggests that IL-17 inhibitors do not appear to be related with TB reactivation and/or TB infection. A recent study pooled data from 28 clinical trials of secukinumab in PsO, PsA, and axSpA with a follow-up period of up to 5-year, encompassing 12,319 patients. Among these, 13 (0.1%) were identified with TBI, of which 7 were considered new cases, with no instances of TBD reported. Isoniazid therapy was documented for 4 of the 7 cases, while information regarding TBI therapy was unavailable for 2 cases, and one patient reported no treatment. The investigation demonstrated that secukinumab is safe and could be even used in PsO patients who are unable to receive TB prophylaxis [33]. Besides, a study using nationwide cohort design demonstrated that IL-17 inhibitors may be advantageous considering TB infection compared to TNF antagonist [61].

This study has limitations due to its retrospective design and limited sample size, with data incompleteness arising from the potential absence of individual or multiple entries. Since patients with axSpA, PsA, and PsO are referred to and treated at specialized centers, the quality and completeness of the collected data heavily relied on the detail and accuracy of the medical charts maintained at these sites. Additionally, the lack of standardized reporting on medical charts for disease screening and monitoring posed common challenges in this type of study, such as inconsistent data collection. Furthermore, the small sample size may restrict the extrapolation of our findings to a wider patient demographic. One important limitation of this study is the absence of a control group, which restricts the ability to draw causal inferences regarding treatment effects. However, this limitation is partially mitigated by the longitudinal design, which included follow-up assessments both before and after the initiation of secukinumab. This within-subject comparison allows for the evaluation of changes over time and provides meaningful insights into treatment patterns and outcomes in routine clinical practice, thereby enhancing the interpretability and relevance of the findings. Despite these limitations, the safety profile of secukinumab has shown no increase in TB cases, even in a country with a high TB prevalence – a finding expected by the authors [33, 62, 63].

## Conclusion

The current real-world study in Brazil evaluates the incidence of TBD cases in patients with axSpA, PsA and PsO treated with secukinumab. No TBD cases were recorded during the study. The majority of the patients were negative for TBI after receiving secukinumab. Further studies are recommended to increase the understanding and prevent TB among axSpA, PsO, and PsA patients.

**Table 1:** Demographic characteristics according to psoriatic disease subgroup

	axSpA	PsA	PsO	axSpA + PsA	axSpA + PsO	Overall *(n = 152)*
**Primary diagnosis n(%)**	30 (19.7%)	90 (59.2%)	29 (19.1%)	2 (1.3%)	1 (0.7%)	-
**Mean (SD) disease duration (months)**	93.6 (55.3)	113.7 (80.7)	184.6 (113.7)	47.5 (21.4)	77.6 (-)	-
Age* (years)						
Mean (SD)	45.1 (11.2)	53.5 (13.5)	53.6 (13.0)	54.0 (2.8)	53.0 (-)	51.8 (13.2)
**Gender at birth -** n (%)						
Male	14 (46.7%)	46 (51.1%)	15 (51.7%)	1 (50.0%)	1 (100.0%)	77 (50.7%)
**Ethnicity** - n (%)						
White	29 (96.7%)	71 (78.9%)	23 (88.5%)	2 (100.0%)	1 (100.0%)	126 (84.6%)
Mixed	0 (-)	15 (16.7%)	2 (7.7%)	0 (-)	0 (-)	17 (11.4%)
Black	0 (-)	2 (2.2%)	1 (3.8%)	0 (-)	0 (-)	3 (2.0%)
Asian	0 (-)	1 (1.1%)	0 (-)	0 (-)	0 (-)	1 (0.7%)
Indigenous	0 (-)	0 (-)	0 (-)	0 (-)	0 (-)	0 (-)
Other	1 (3.3%)	1 (1.1%)	0 (-)	0 (-)	0 (-)	2 (1.3%)

axSpA, axial spondyloarthritis; PsA, psoriatic arthritis; PsO, psoriasis; SD, standard deviation

*Age at study enrollment; Patients with PsA + PsO were considered as PsA

Missing values: Ethnicity, PsO (3)

**Table 2:** Comorbidities according to psoriatic disease subgroup

	axSpA (*n* = 30)	PsA (*n* = 90)	PsO (*n* = 29)	axSpA + PsA (*n* = 2)	axSpA + PsO (*n* = 1)	Overall (*n* = 152)
**Comorbidity up to secukinumab initiation, n (%)**						
Yes	24 (80.0%)	57 (63.3%)	16 (55.2%)	1 (50.0%)	1 (100.0%)	99 (65.1%)
**Comorbidities**,** n (%)**						
N of comorbidities	50	135	49	1	4	239
Hypertension	6 (12.0%)	16 (11.9%)	8 (16.3%)	0 (-)	1 (25.0%)	31 (13.0%)
Diabetes mellitus	3 (6.0%)	12 (8.9%)	5 (10.2%)	1 (100.0%)	0 (-)	21 (8.8%)
Dyslipidemia	2 (4.0%)	12 (8.9%)	4 (8.2%)	0 (-)	0 (-)	18 (7.5%)
Fibromyalgia	8 (16.0%)	7 (5.2%)	0 (-)	0 (-)	0 (-)	15 (6.3%)
Depression	4 (8.0%)	9 (6.7%)	2 (4.1%)	0 (-)	0 (-)	15 (6.3%)
Anxiety	2 (4.0%)	8 (5.9%)	4 (8.2%)	0 (-)	0 (-)	14 (5.9%)
Obesity	1 (2.0%)	8 (5.9%)	1 (2.0%)	0 (-)	0 (-)	10 (4.2%)
Hypothyroidism	3 (6.0%)	5 (3.7%)	2 (4.1%)	0 (-)	0 (-)	10 (4.2%)
Gastroesophageal reflux disease	2 (4.0%)	1 (0.7%)	1 (2.0%)	0 (-)	0 (-)	4 (1.7%)
Hepatic steatosis	0 (-)	3 (2.2%)	1 (2.0%)	0 (-)	0 (-)	4 (1.7%)
Hepatitis B	0 (-)	4 (3.0%)	0 (-)	0 (-)	0 (-)	4 (1.7%)
Hypertriglyceridemia	0 (-)	3 (2.2%)	0 (-)	0 (-)	0 (-)	3 (1.3%)
Osteoporosis	1 (2.0%)	2 (1.5%)	0 (-)	0 (-)	0 (-)	3 (1.3%)
Vitamin D deficiency	2 (4.0%)	1 (0.7%)	0 (-)	0 (-)	0 (-)	3 (1.3%)
Bipolar disorder	0 (-)	2 (1.5%)	0 (-)	0 (-)	0 (-)	2 (0.8%)
Epilepsy	0 (-)	1 (0.7%)	1 (2.0%)	0 (-)	0 (-)	2 (0.8%)
Gastritis	1 (2.0%)	1 (0.7%)	0 (-)	0 (-)	0 (-)	2 (0.8%)
Liver disorder	0 (-)	0 (-)	2 (4.1%)	0 (-)	0 (-)	2 (0.8%)
Malignant melanoma	1 (2.0%)	0 (-)	1 (2.0%)	0 (-)	0 (-)	2 (0.8%)
Osteoarthritis	0 (-)	2 (1.5%)	0 (-)	0 (-)	0 (-)	2 (0.8%)
Other*	14 (28.0%)	38 (28.2%)	17 (34.7%)	0 (-)	3 (75.0%)	72 (30.1%)

axSpA, axial spondyloarthritis; PsA, psoriatic arthritis; PsO, psoriasis. * Other comorbidities appeared only once

Note: One patient could have more than one comorbidity; Patients with PsA + PsO were considered as PsA

**Table 3:** Concomitant medications before and after Secukinumab initiation

Variables	Prior Secukinumab initiation	After secukinumab initiation
**Number of concomitant medication**, n	482	120
Mean number of medication by patient	3.35	2.26
N of patients	144	53
**axSpA PsA PsO-target or prophylaxis medications**,** n** by patient	123 (85.4%)	17 (32.0%)
**DMARDs (Disease-Modifying Antirheumatic drug)**,** n (%)**	80 (65.0%)	1 (5.9%)
Methotrexate oral	74 (60.2%)	1 (5.9%)
Mean (SD) dosage (mg)	18.1 (10.0)	20.0 (-)
Median [Q1;Q3] dosage (mg)	15.0 [15.0; 20.0]	20.0 [20.0;20.0]
** Immunobiologics**,** n (%)**	72 (58.5%)	0 (-)
** Corticosteroids**,** n (%)**	17 (13.8%)	7 (41.2%)
** NSAIDs (Non-steroidal Anti-inflammatory drugs)**,** n (%)**	10 (8.1%)	3 (17.6%)
** Other**	48 (39.0%)	12 (70.6%)

Missing: 22 medications were not included in the table due to the absence of start date

DMARDs: Methotrexate oral (74; 1), Methotrexate subcutaneos (1; 0), Cyclosporin (8; 0), Leflunomide (4; 0), Sulfasalazine (7; 0), Acitretin (20; 0), Upadacitinib (1; 0), Abatacept (1; 0)

Immunobiologics: Infliximab (6; 0), Etanercept (30; 0), Adalimumab (52; 0), Golimumab (3; 0), Guselkumab (1; 0), Certolizumab pegol (2; 0), Ustekinumab (3; 0)

Corticosteroids: Prednisone (2; 0), Betamethasone (4; 4), Dexamethasone (0; 1), Clobetasol (10; 2), Clobetasol propionate (2; 2), Deflazacort (1; 0), Corticosteroids, topical (1; 0), Ulobetasol (1; 0)

NSAIDs: Nimesulide (0; 1), Celecoxib (2; 1), Etoricoxib (1; 0), Ibuprofen (1; 0), Ketoprofen (0; 1), Meloxicam (1; 0), Naproxen (4; 0), Naproxen sodium (1; 0), Esomeprazole + Naproxen (1; 0)

Q1: first quartile; Q3: third quartile

Other: Topical Agents/Emollients, Keratolytics/Dermatological Agents, Analgesics/Antipyretics, Vitamins & Supplements, Muscle Relaxants, Gastrointestinal Agents, NSAIDs (Non-Steroidal Anti-Inflammatory Drugs), Iron Supplements and Antibiotics/Antimicrobials

**Table 4:** Secukinumab treatment regimen

Variables	All patients (*n* = 152)
**Secukinumab treatment duration (months)**	
Mean (SD)	39.3 (13.8)
Median [Q1; Q3]	35.8 [28.3;46.9]
Minimum/Maximum	24.0 / 84.2
**Initial dosage**	
**Loading dosage prescribed - n (%)**	141 (92.8%)
**Dose**,** n (%)**	
150 mg	31 (22.0%)
300 mg	110 (78.0%)
**Maintenance dosage**	
**Dose**,** n (%)**	
150 mg	35 (23.0%)
300 mg	117 (77.0%)
**Interruption of secukinumab treatment n (%)**	18 (11.8%)
Interruption duration** (months)	
Mean (SD)	3.8 (3.1)
Median [Q1; Q3]	2.0 [1.9;5.5]
Minimum / Maximum	1.1 / 13.0
**Immunobiologic use before secukinumab initiation n (%)**	72 (47.4%)

Q1, first quartile; Q3: third quartile; SD, standard deviation

**Interruption duration (months) = interruption date of secukinumab – restart date of secukinumab

**Table 5:** Active and LTBI before and after Secukinumab per Imuune mediated disease

	axSpA (*n* = 28)	PsA (*n* = 89)	PsO (*n* = 28)	axSpA + PsA (*n* = 2)	axSpA + PsO (*n* = 1)	Overall (*n* = 148)
**Active tuberculosis cases**,** n**	0	0	0	0	0	0
**Prior secukinumab initiation**						
Tuberculosis test for latent Tuberculosis, n (%)						
Negative	25 (89.3%)	70 (78.7%)	19 (67.9%)	1 (50.0%)	1 (100.0%)	116
Positive	3 (10.7%)	19 (21.3%)	9 (32.1%)	1 (50.0%)	0 (-)	32
Prophylactic antibiotic therapy among those who tested positive, n (%)						
No	1 (33.3%)	5 (26.3%)	5 (55.6%)	0 (-)	0 (-)	11
Yes	2 (66.7%)	14 (73.7%)	4 (44.4%)	1 (100.0%)	0 (-)	21
**After secukinumab initiation**						
Tuberculosis test for latent Tuberculosis, n (%)						
Negative	28 (100.0%)	86 (96.6%)	26 (92.9%)	2 (100.0%)	1 (100.0%)	143
Positive	0 (-)	3 (3.4%)	2 (7.1%)	0 (-)	0 (-)	5
Prophylactic antibiotic therapy among those who tested positive, n (%)						
No	0 (-)	0 (-)	0 (-)	0 (-)	0 (-)	0
Yes	0 (-)	3 (100.0%)	2 (100.0%)	0 (-)	0 (-)	5
N of participants not tested for TB prior to secukinumab initiation	2	5	5	0	0	12
Patient not tested for TB prior Secukinumab initiation with prophylactic antibiotic use, n (%)	-	1 (8.3%)	0 (-)	-	-	1

axSpA, axial spondyloarthritis; PsA, psoriatic arthritis; PsO, psoriasis; TB, tuberculosis

Note: Prophylactic antibiotic had considered only oral rifampicin or isoniazid

## Data Availability

No datasets were generated or analysed during the current study.
